# Correction: Matriptase drives dissemination of ovarian cancer spheroids by a PAR-2/PI3K/Akt/MMP9 signaling axis

**DOI:** 10.1083/jcb.20220911412102025c

**Published:** 2025-12-19

**Authors:** Nisha R. Pawar, Marguerite S. Buzza, Nadire Duru, Amando A. Strong, Toni M. Antalis

Vol. 222, No. 11 | https://doi.org/10.1083/jcb.202209114 | September 22, 2023

The authors regret that, during figure assembly, the same image was mistakenly placed in Figure 4 C (ES2-Mat / GB-83) and Figure 4 D (ADR-Res-Mat / GB-83). The original and corrected Figure 4 are shown here. This error does not affect the conclusions of the study, and the figure legend remains unchanged.

This error appears in print and in PDFs downloaded before December 4, 2025. The authors apologize for any confusion this may have caused.

**Figure 1:**
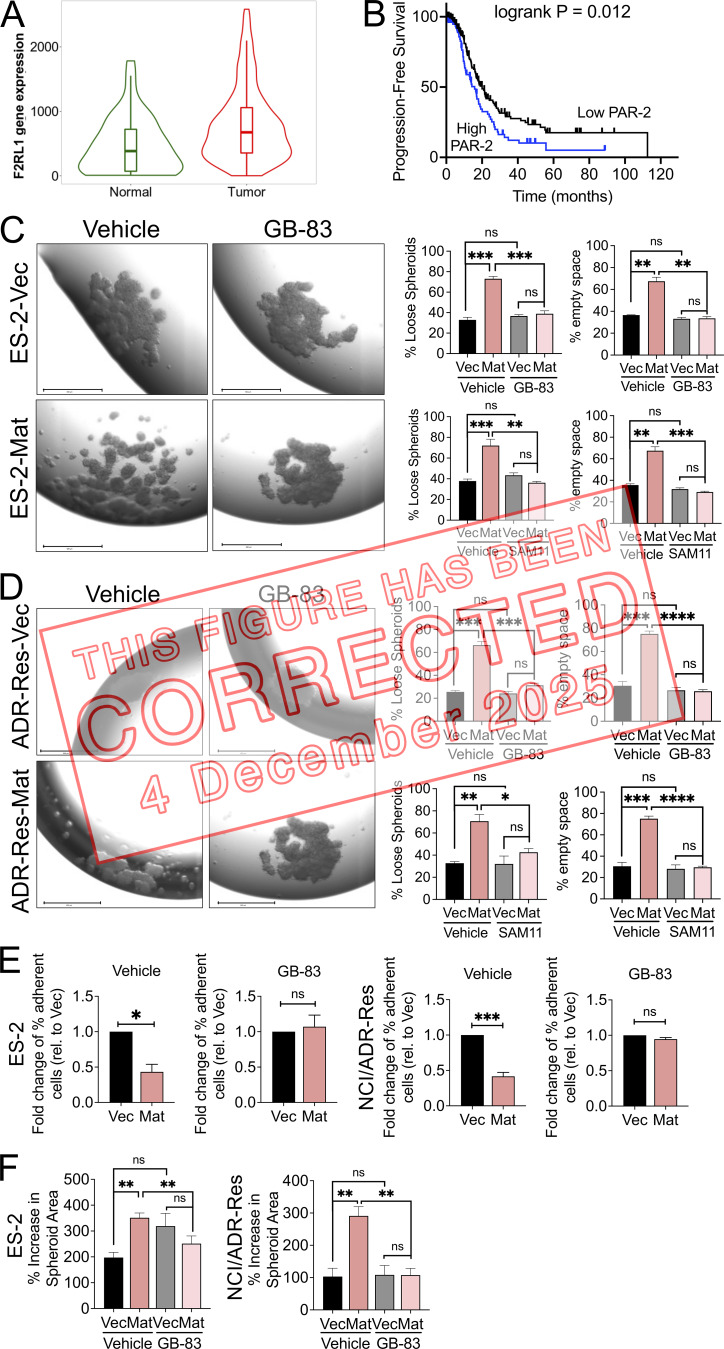


**Figure 2:**
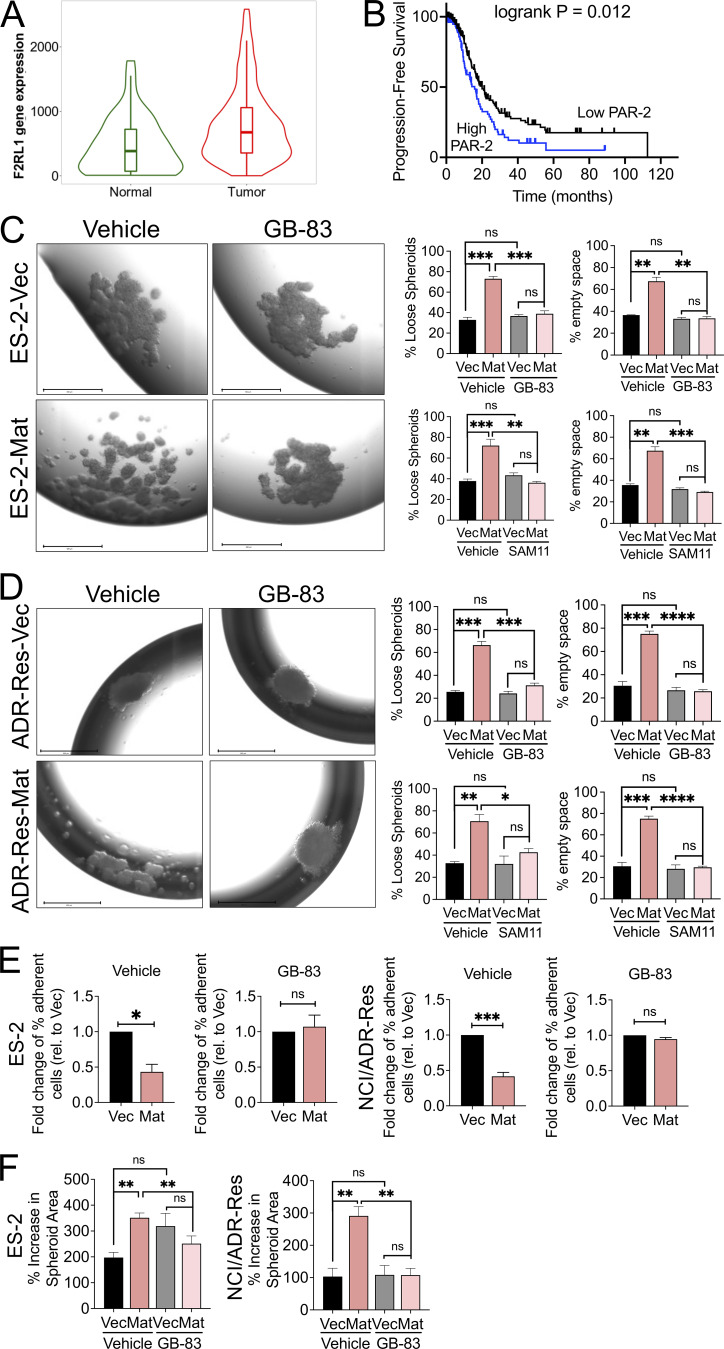
**Matriptase-mediated loose spheroid morphology is attenuated by PAR-2 inhibition.** **(A)** Analysis of PAR-2 mRNA expression amongst normal ovary tissue (*n* = 46) and ovarian tumors (*n* = 744) using publicly available gene chip data obtained from TNM Plotter (P = 1.54 e^−06^). **(B)** Kaplan–Meier progression-free survival analysis of a cohort of stage III and IV OvCa patients with high and low PAR-2 expression using publicly available data obtained from KMPlotter (outlier arrays excluded; *n* = 220; logrank P = 0.012). **(C and D)** ES-2 and NCI/ADR-Res Vec or Mat hanging-drop spheroids were formed in the presence of vehicle control (DMSO), GB-83 (5 and 25 µM respectively), or SAM11 (2 ng/ml and 1 µg/ml, respectively) for 4 d. Spheroids were visualized by EVOS (representative images shown at 4× magnification, scale bars represent 650 µm in left image panels) and morphology was assessed under each condition. Graphs show the quantitation of the spheroid morphology (% loose spheroids and % empty space) as described in the Materials and methods. Data represents the average of three independent experiments (error bars are ± SEM; *P < 0.05, **P < 0.01, ***P < 0.005, ****P < 0.001, ns = not significant, two-tailed unpaired Student’s *t* tests). **(E)** Homotypic cell–cell interactions. ES-2 and NCI/ADR-Res Vec and Mat cells labeled with CellTrackerOrange were seeded onto respective homotypic monolayers in the presence of vehicle control (DMSO) or GB-83 (25 µM); fluorescence was measured after 1 h of adhesion, before and after wash with PBS. Fold change of remaining fluorescence relative to Vec is represented as an average of three independent experiments performed with at least three replicates each (error bars are ± SEM; *P < 0.05, ***P < 0.005, ns = not significant, two-tailed unpaired Student’s *t* tests). **(F)** Spheroid disaggregation. ES-2 and NCI/ADR-Res Vec and Mat spheroids generated on agarose hydrogel and labeled with calcein-AM were seeded onto LP9 mesothelial monolayers and disaggregation in the presence of vehicle control (DMSO) or GB-83 (25 µM) was monitored over 72 h by EVOS. The percentage increase in spheroid area was measured by ImageJ and represented as an average of three independent experiments performed in triplicate (error bars are ± SEM; **P < 0.01, ns = not significant, two-tailed unpaired Student’s t tests).

